# Global trend of food‐induced anaphylaxis: Up to date

**DOI:** 10.1111/pai.70246

**Published:** 2025-12-02

**Authors:** Agnes Sze‐yin Leung, Elizabeth Estrada‐Reyes, Kaito Goto, Chiung‐Hui Huang, Janice Min Li, Sowmya Arudi Nagarajan, Thushali Ranasinghe, Sakura Sato, Witchaya Srisuwatchari, Benjamin Zepeda‐Ortega, Elizabeth Huiwen Tham

**Affiliations:** ^1^ Department of Paediatrics, Faculty of Medicine, Prince of Wales Hospital The Chinese University of Hong Kong Shatin Hong Kong; ^2^ Hong Kong Hub of Paediatric Excellence (HOPE) The Chinese University of Hong Kong Shatin Hong Kong; ^3^ Department of Pediatric Clinical Allergology and Immunology Hospital Angeles Metropolitano Mexico City Mexico; ^4^ Department of Allergy, Clinical Research Center for Allergy and Rheumatology NHO Sagamihara National Hospital Sagamihara Kanagawa Japan; ^5^ Department of Pediatrics St. Marianna University Kawasaki Kanagawa Japan; ^6^ Department of Paediatrics, Yong Loo Lin School of Medicine National University of Singapore Singapore; ^7^ Department of Pediatrics Motherhood Hospitals Bengaluru India; ^8^ Department of Immunology and Molecular Medicine, Faculty of Medical Sciences University of Sri Jayewardenepura Nugegoda Sri Lanka; ^9^ Institute of Allergology and Immunology University of Sri Jayewardenepura Nugegoda Sri Lanka; ^10^ Division of Allergy and Immunology, Department of Pediatrics, Faculty of Medicine Siriraj Hospital Mahidol University Bangkok Thailand; ^11^ Private Practice, Pediatric Allergology, Toluca Mexico City Mexico

**Keywords:** adrenaline autoinjector, allergen labelling, allergen patterns, food‐induced anaphylaxis, geographic variation, global epidemiology, novel allergens

## Abstract

This review examines the evolving global landscape of food‐induced anaphylaxis (FIA), revealing critical epidemiological shifts that challenge traditional allergen management paradigms. As the leading cause of anaphylaxis worldwide, FIA demonstrates striking geographic, age‐specific, and temporal patterns that reflect broader changes in dietary practices, environmental exposures, and food production systems. Emerging trends reveal novel allergen sources that evade current regulatory frameworks, including non‐priority legumes, edible insects and galacto‐oligosaccharides with distinct cross‐reactivity patterns and the expanding prevalence of buckwheat, tree nut and seeds anaphylaxis. Complex syndromes such as lipid transfer protein syndrome, tick‐borne α‐Gal syndrome and food‐dependent exercise‐induced anaphylaxis represent diagnostic challenges requiring heightened clinical suspicion. Critical knowledge gaps remain in certain regions where limited surveillance, inadequate adrenaline access, and uncharacterized regional allergens create substantial management disparities. The review emphasizes urgent needs for comprehensive global surveillance networks, precision diagnostic tools for atypical presentations, expanded allergen labeling policies encompassing novel proteins, and equity‐centered interventions addressing geographic disparities in emergency treatment access.

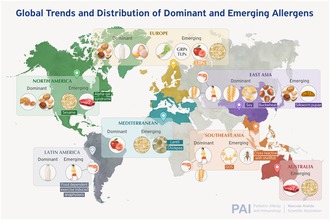

## Graphical abstract

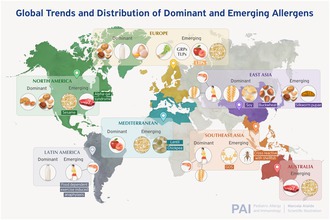

**Abbreviations**
AAIs: Adrenaline Autoinjectors
AGS: Alpha‐Gal Syndrome
APRA: Asia‐Pacific Research Network for Anaphylaxis
AVN: Allergy‐Vigilance Network (Réseau d'Allergo‐Vigilance, RAV)
BAT: basophil activation test
EFA: European Federation of Allergy and Airways Diseases Patients' Associations
FA: food allergy
FARE: Food Allergy Research and Education
GA^2^LEN: Global Allergy and Asthma European Network
GOS: galacto‐oligosaccharides
GRPs: Gibberellin‐regulated proteins
LTP: lipid Transfer Protein
NORA: Network for Online Registration of Anaphylaxis
nsLTPs: non‐specific lipid transfer proteins
PFAS: Pollen‐Food Allergy Syndrome
RAV: Réseau d'Allergo‐Vigilance (Allergy‐Vigilance Network, AVN)
SDEIA: Soy‐Dependent Exercise‐Induced Anaphylaxis
SPT: skin prick test
TLPs: Thaumatin‐like proteins
vGOS: Vivinal® GOS (commercial galacto‐oligosaccharide formulation)

**Key message**

This review highlights critical shifts in global food‐induced anaphylaxis patterns and identifies emerging allergens including specific legumes, edible insects, and galacto‐oligosaccharides. Complex syndromes such as lipid transfer protein syndrome, tick‐borne α‐Gal syndrome, and food‐dependent exercise‐induced anaphylaxis present diagnostic challenges requiring heightened clinical awareness. The review emphasizes the need for improved surveillance, updated labeling policies, equitable treatment access, and targeted research.

## 1 GLOBAL PERSPECTIVE ON FOOD‐INDUCED ANAPHYLAXIS

Food‐induced anaphylaxis (FIA), a potentially fatal systemic reaction, is the leading cause of anaphylaxis globally, especially in children.^1^ The global burden of food allergy (FA) has been increasing over the past decades. Studies have indicated an increase in FA prevalence^2^, ^3^, ^4^, ^5^, ^6^, ^7^ and FIA incidence^8^ in many Western countries, and a similar trend has also emerged in the Eastern continent in the past decade.^9^, ^10^, ^11^

The reported anaphylaxis‐related case fatality rate was 0.7%–2% in the early 21st century.^12^, ^13^ However, with improved allergy vigilance systems and enhanced healthcare resources, this rate has substantially declined. While contemporary case fatality rates in high‐income countries are low (<1% of hospital admissions; <1 per million annually).^14^, ^15^, ^16^ Data from the United Kingdom spanning two decades (1998–2018) illustrate improving outcomes, with confirmed fatal food anaphylaxis declining from 0.7% to 0.19% of hospital admissions.^17^ In the United States, large‐scale epidemiological studies report case fatality rates of 0.25%–0.33% among hospitalized patients and emergency department visits, with the vast majority of anaphylaxis cases achieving favorable outcomes.^18^ Key risk factors include adolescence/young adulthood, uncontrolled asthma, prior anaphylaxis, and delayed adrenaline administration.^1^, ^14^, ^17^, ^19^, ^20^ Peanuts, tree nuts, and cow's milk are predominant fatal triggers in well‐studied regions.^14^, ^17^

### 1.1 The global knowledge gap

Current data predominantly reflect the experience of high‐income and English‐speaking countries with established healthcare infrastructure and standardized anaphylaxis management protocols. A significant knowledge gap exists regarding the anaphylaxis burden in emerging economies and low‐resource settings, where multiple factors may substantially alter both the epidemiology and outcomes of FIA.

In non‐English speaking countries, the landscape of FIA presents fundamentally different challenges: region‐specific allergens, limited access to emergency care, scarce allergy services, critical shortages of adrenaline auto‐injectors, and potentially rising FIA incidence due to dietary shifts.^21^, ^22^ The convergence of increasing anaphylaxis incidence in these regions with inadequate management resources creates an alarming blind spot in global anaphylaxis epidemiology.

This review aims to illuminate the global pattern of FIA, expanding beyond the well‐documented Western experience to explore emerging allergens worldwide and the diverse triggers that reflect local dietary patterns and culture. Furthermore, it seeks to identify and address the critical gaps in anaphylaxis management that persist in resource‐limited settings, proposing strategies to bridge these disparities and improve outcomes for patients with FIA across all healthcare environments.

### 1.2 Global pattern of anaphylaxis triggers

Food‐induced anaphylaxis exhibits remarkable heterogeneity across three critical dimensions: patient age, geographic location, and temporal evolution, reflecting the interplay between developmental immunology, regional dietary patterns, and the evolution of global food systems.

#### 1.2.1 Age‐related patterns

The spectrum of food allergens and FIA shifts dramatically with age, as shown in Table S1. During early childhood (0–3 years), cow's milk and hen's egg consistently emerge as the predominant triggers across all geographic regions. European data (NORA) attribute 12% of anaphylaxis to milk and 14% to egg, while Latin American studies attribute a substantially higher proportion (38.7%) to milk.^23^, ^24^ Asia‐Pacific registry data (APRA) show egg dominance (31.5%) followed by milk (22.3%) in this age group.^21^ During middle childhood (4–12 years), a notable epidemiological shift occurs during this developmental period, characterized by declining milk and egg prevalence concurrent with increasing peanut and tree nut allergies. Asia‐Pacific data demonstrate this transition clearly: tree nuts (27.3%) and shellfish (18.2%) emerge at ages 4–6, with shellfish becoming dominant (37.1%) by ages 7–12.^21^ Regional variations include an increased incidence of wheat‐induced anaphylaxis in East Asian populations.^21^ Among adolescents aged 13 and 17 years, shellfish emerge as the primary trigger, particularly in coastal and Asian regions, representing 40.7% of cases in Asia‐Pacific adolescent populations.^21^, ^25^ Peanuts and tree nuts remain significant (18.6%), while milk and egg are rarely primary triggers in this age group.

#### 1.2.2 Geographic influences

Geographic factors, alongside age, influence regional anaphylaxis patterns (Table S1). In Europe, tree nuts, particularly cashews, hazelnuts, and walnuts, constitute major triggers alongside traditional milk and egg allergens.^23^, ^26^, ^27^ Unique regional allergens include celery, often associated with pollen‐food allergy syndrome (PFAS), which comprises 16% of the “other tree nuts/vegetables” category in NORA data^23^

However, in North America, peanut allergy demonstrates exceptional prominence across all pediatric age groups, with significant temporal increases in emergency department presentations.^23^, ^28^ Tree nuts, particularly cashew and walnut, represent major triggers. Emergency department data (2005–2014) reveal substantial increases in peanut, tree nut, and fruit/vegetable anaphylaxis across all pediatric demographics.^29^, ^30^

In Latin America, cow's milk dominates across all pediatric age groups, representing 38.7% of triggers in the 0–17 year population and showing minimal age‐related decline compared to other regions.^24^, ^31^

While in Asia, distinct regional allergen profiles emerge, including wheat and buckwheat allergies in East Asian populations (Japan: wheat 20%, buckwheat 1.5%).^21^, ^32^, ^33^ Unique triggers include fish roe, bird's nest, and significant shellfish‐induced anaphylaxis in adolescent populations.^33^, ^34^ Peanut‐induced anaphylaxis, although present, has a lower prevalence compared to Western populations. GOS‐induced anaphylaxis is a distinctly regional phenomenon, documented in atopic individuals within Southeast Asia (particularly Singapore).^35^

In terms of fatal food anaphylaxis, peanut and tree nuts consistently emerge as the leading causes across all reported regions.^17^ Cow's milk is another major contributor, particularly among children, with a strikingly high proportion of fatalities reported in the United Kingdom (26%) and France (31%). In Australia and the United States, shrimp and other crustaceans (29%–45%) are prominent triggers of fatal reactions. Notably, fatalities can also result from less commonly recognized allergens, which vary by region—for example, sesame in the United Kingdom (0.8%–3%) and buckwheat (5%)—highlighting the importance of region‐specific surveillance and allergen labeling policies.

#### 1.2.3 Temporal evolution

**FIGURE 1:**
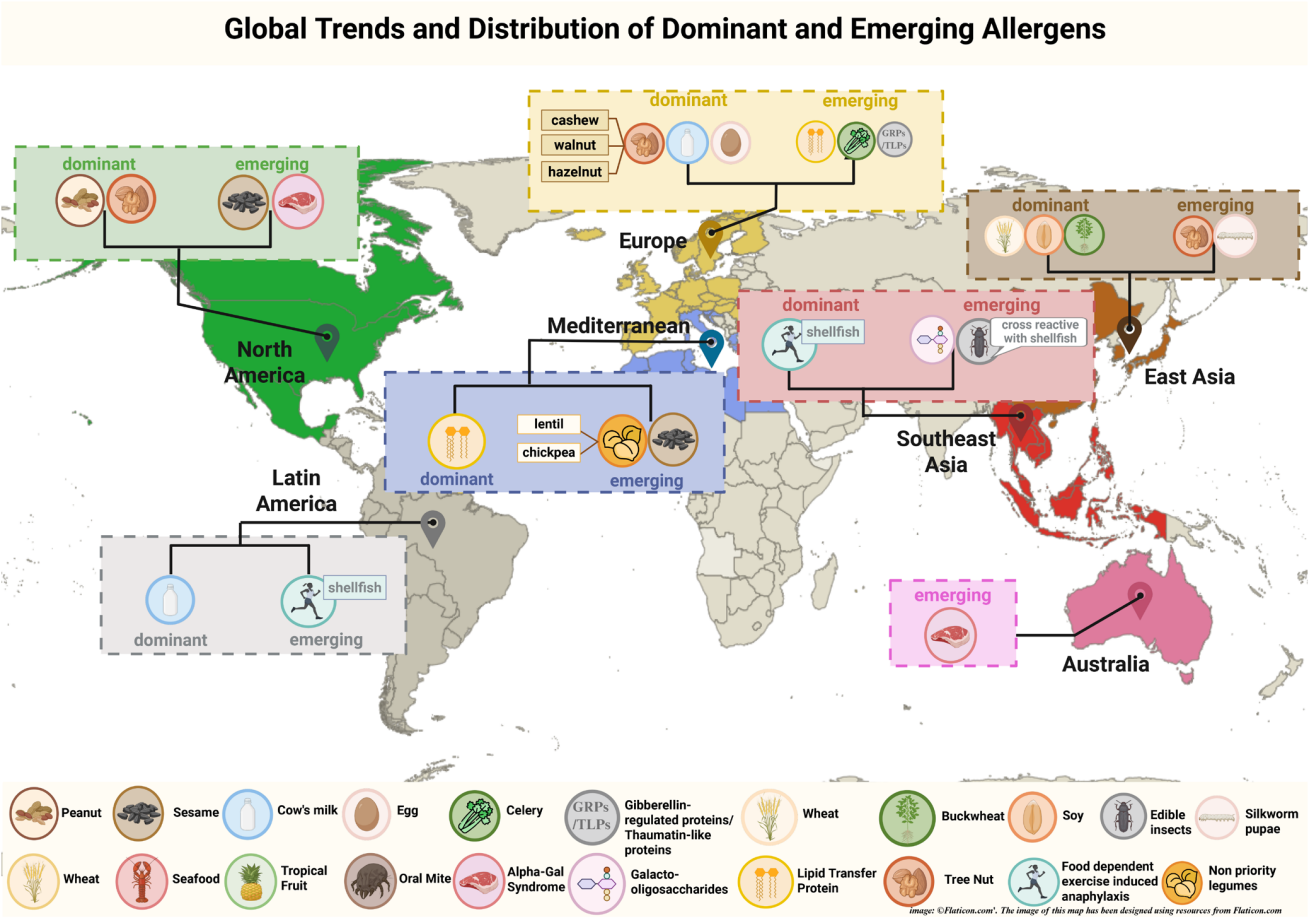
Global landscape of food allergens: Dominant triggers and emerging allergens by region. Map‐style summary showing the principal food triggers of anaphylaxis by region and notable emerging allergens. “Dominant allergens” denote established high‐burden triggers; “Emerging allergens” reflect allergens with increasing reports or newly recognized syndromes. The Mediterranean panel highlights lipid transfer protein (LTP) syndrome; Europe also notes gibberellin‐regulated proteins (GRPs) and thaumatin‐like proteins (TLPs). North America emphasizes alpha‐gal syndrome (delayed red‐meat allergy linked to tick exposure). Southeast Asia includes food‐dependent exercise‐induced anaphylaxis (FDEIA) predominantly from shellfish and rising reports related to edible insects; East Asia highlights buckwheat and increasing tree‐nut reactions. alpha‐gal, galactose‐α‐1,3‐galactose; FDEIA, food‐dependent exercise‐induced anaphylaxis; GOS, galacto‐oligosaccharides; GRP, gibberellin‐regulated protein; LTP, lipid transfer protein; TLP, thaumatin‐like protein. Regional examples are illustrative and not exhaustive; heterogeneity within regions is expected.

The temporal evolution of FIA reveals distinct emerging patterns that warrant detailed examination. Novel protein sources such as those from plant matrices—peanut, tree nuts (like cashew), sesame, soy, other legumes (notably pea, chickpea, and lentil), and buckwheat—and edible insects are increasingly recognized as allergens causing FIA in adults and children worldwide. There is regional variation in their prevalence and ranking, but each exceeds the 1% threshold in population‐based studies and systematic reviews.^36^ Pollen‐food allergy syndrome, specifically Lipid transfer protein (LTP) allergy, shows a clear northward expansion across Europe, evolving from a Mediterranean phenomenon to an increasingly recognized cause of severe anaphylaxis in Northern European populations.^37^ Alpha‐gal syndrome represents a novel paradigm of tick‐mediated mammalian meat allergy, demonstrating significant geographic variation with dramatic increases in North America but less significant clinical impact in Asia.^38^ However, the temporal dimension may reflect improved recognition and reporting, as many apparent increases in specific allergens may partly result from enhanced awareness and better diagnostic capabilities, in addition to epidemiological changes. These emerging anaphylaxis triggers are highlighted in Figure 1 and will be examined in detail below to elucidate their distinct epidemiological patterns, underlying mechanisms, and clinical significance.

## 2 THE EVOLVING PROFILE OF PLANT‐BASED FOOD‐INDUCED ANAPHYLAXIS

Novel foods represent a paradigmatic shift in global nutrition, offering sustainable, nutrient‐dense alternatives to conventional dietary sources.^39^ The Food and Agriculture Organization and World Health Organization (FAO/WHO) define novel foods as those previously absent from widespread human consumption, often emerging through technological innovation and biotechnological advancement.^40^ This category encompasses an expanding spectrum of food sources, including plant‐based alternatives,^41^ entomophagy products,^42^ cultivated meat,^43^ three‐dimensionally printed foods,^44^ microbial fermentation‐derived ingredients,^45^ algae‐based products,^46^ and nanotechnology‐enhanced foods.^47^ The introduction of novel foods into mainstream diets presents unprecedented challenges in allergenicity assessment.

### 2.1 Legume anaphylaxis

Among plant proteins, particularly those derived from legumes, cereals, pseudocereals (quinoa, amaranth, buckwheat, chia), and oilseeds (rapeseed, pumpkin, hemp), represent the most promising sustainable protein alternatives for future food systems. Among these, legumes are the most common triggers of anaphylaxis, mostly caused by peanut and soybean, with increasing recognition of non‐priority legumes (lentil, chickpea, pea, lupine), especially in regions with high legume consumption.^37^, ^48^, ^49^

Legume‐induced anaphylaxis exhibits geographical heterogeneity reflecting regional dietary patterns and agricultural practices, with peanut representing the predominant trigger in Western populations (affecting 25% of food‐allergic children with 0.2%–2% prevalence in US/Europe/Australia),^2^, ^3^, ^50^, ^51^, ^52^ while soy allergy predominates in East Asia^33^, ^53^, ^54^ and lentil/chickpea allergies dominate Mediterranean and South Asian regions (80% and 59%, respectively among early‐fed infants,^55^ with 7.7% prevalence in Indian children^56^).

Cross‐reactivity patterns complicate clinical management, with up to 60% of peanut‐allergic children demonstrating sensitization to additional legumes, particularly fenugreek, lentils, and peas, while lupine allergy presents unique challenges through 15%–20% cross‐reactivity with peanuts and frequent occurrence as hidden allergens in processed foods,^57^ contributing to 0.8%–2.3% of European anaphylaxis cases.^58^, ^59^ Despite well‐documented serological cross‐reactivity between peanut and other legumes, clinical relevance varies significantly, making the diagnosis of true allergy complex and reliant on resource‐intensive oral challenges or alternatives like SPTs, which are further complicated by regional limitations in standardized reagent availability often necessitating less reliable prick‐prick testing.^48^, ^60^

### 2.2 Buckwheat anaphylaxis

Buckwheat‐induced anaphylaxis represents an emerging clinical concern, with increasing incidence correlating to buckwheat's expanded utilization as a gluten‐free, high‐protein ingredient in vegetarian diets and novel plant‐based products. The allergenicity profile encompasses multiple potent storage proteins (Fag e 1, Fag e 2, Fag e 3, Fag e 5, Fag t 6) capable of eliciting severe IgE‐mediated reactions following minimal exposure.^61^, ^62^, ^63^, ^64^

Buckwheat‐induced anaphylaxis demonstrates geographical variation in epidemiological burden, with endemic prevalence in East Asia contrasting sharply with sporadic cases in Western populations. Korean pediatric cohorts exhibit the highest incidence rates (6.5% of food anaphylaxis cases, *n* = 740),^65^ while Japanese schoolchildren demonstrate population‐level prevalence of 0.004% (4.3/100,000),^66^ compared to negligible rates in European populations (0.01/100,000 person‐years in Finland,^67^ 0.1% in Italy^68^). Clinical presentations are characterized by rapid onset within 1 h post‐exposure, with documented fatal cases associated with traditional foods such as Korean “nyan‐mien” noodles and Japanese “zaru soba,” particularly when exercise serves as a cofactor.^69^, ^70^ Atypical manifestations include biphasic reactions requiring extended monitoring (12–24 h) and cross‐reactivity with poppy seeds,^71^ peanuts,^72^ quinoa,^73^ coconuts,^74^ and latex.^75^, ^76^

### 2.3 Seed anaphylaxis

The United States has mandated sesame labeling as a major food allergen in 2023, aligning with the European Union, Australia, and Canada—a policy shift reflecting its emergence as the predominant seed allergen,^77^, ^78^ affecting 0.1%–0.2% of most English‐speaking populations and up to 0.9% in Middle Eastern regions with high sesame consumption.^79^, ^80^ Sesame demonstrates exceptional allergenic potency, with 38% of allergic individuals experiencing severe reactions requiring adrenaline treatment, and exhibits high persistence rates (70%–80% into adulthood) and frequent co‐allergy with peanuts or tree nuts (>50%).^79^, ^80^, ^81^ Sunflower seed allergy, while less prevalent (≤0.38% in pediatric populations), shows increasing incidence with 25% of affected children experiencing anaphylaxis, typically presenting in early childhood.^82^ Additional seeds, including poppy, pumpkin, and fenugreek, demonstrate a lower allergenic frequency but retain anaphylactic potential, with documented severe reactions.^83^

### 2.4 Tree nut anaphylaxis

Nut allergies exemplify a notable temporal evolution: historically rare in East Asian countries like Japan and South Korea, tree nut anaphylaxis has surged since 2010. Japan demonstrates this transformation most vividly: nuts, once foreign to traditional cuisine, have become mainstream since the 2010s due to wellness trends and global food accessibility. National registry data from facilities certified by the Japanese Society of Allergology confirm that tree nuts, particularly walnuts, have become significant triggers of anaphylaxis in Japanese children.^84^ Hospital‐based and registry studies documented a concurrent rise in tree nut‐induced anaphylaxis.^33^ A regional survey in Aichi Prefecture found that the proportion of FIA cases triggered by tree nuts increased from 6.0% in 2017 to 15% in 2019, making tree nuts one of the most rapidly increasing triggers of anaphylaxis in children.^85^

In a multicenter Korean study, walnut accounted for 41.3% and pine nut for 7.1% of nut/seed‐induced anaphylaxis cases in children, with a mean age of 4.9 years. Anaphylaxis could occur at low levels of specific IgE, and reactions were often severe enough to require emergency care, with adrenaline administered in about half of emergency department cases.^32^ In infants (0–2 years), walnut was the third most common food trigger for anaphylaxis after cow's milk and egg, accounting for 8.3% of cases, with other nuts (excluding walnut and peanut) responsible for 3%.^86^ Population‐based data indicate that, among FIA cases presenting to emergency departments, nuts (including tree nuts) are a leading cause in children and adolescents.^87^, ^88^, ^89^

### 2.5 Entomophagy‐induced anaphylaxis

Edible insect allergy represents an emerging clinical phenomenon as arthropod‐derived proteins are increasingly integrated into food systems. Entomophagy‐induced anaphylaxis represents an emerging clinical entity with significant regional variation in incidence rates, ranging from 0.3% to 19.4% of documented food anaphylaxis cases across different populations.^42^, ^90^ Retrospective medical analyses demonstrate that silkworm pupae account for 3.1% of confirmed food allergy cases in adults,^91^, ^92^ with 46.2% of affected patients exhibiting concurrent shellfish allergies, while anaphylaxis registries report insect‐induced reactions in 0.3% (5/1501 cases) to 8.1% (17/209 cases) of food anaphylaxis.^93^, ^94^

Cross‐reactivity constitutes the primary mechanism underlying insect allergy. Two primary risk populations have been identified: individuals with pre‐existing crustacean allergies and those with occupational exposure to edible insects.^95^ The former group demonstrates the highest propensity for severe cross‐reactive anaphylaxis upon initiation of entomophagy. The major insect allergens—tropomyosin and arginine kinase—are conserved pan‐allergens shared with crustaceans and mites, which explains the frequent co‐sensitization patterns.^90^ Up to 95% of insect‐sensitized individuals demonstrate IgE reactivity to mite allergens, with cross‐reactivity persisting despite food processing or thermal treatment.^96^ However, sensitization does not reliably predict clinical allergy, which can complicate diagnosis.^97^

## 3 THE CHANGING LANDSCAPE OF POLLEN FOOD ALLERGY SYNDROME

The contemporary landscape of PFAS has evolved beyond classical Bet v 1‐mediated cross‐reactivity to encompass diverse allergenic protein families, including gibberellin‐regulated proteins (GRPs),^98^ thaumatin‐like proteins (TLPs),^98^, ^99^ and lipid transfer proteins (LTPs) syndrome,^100^ each presenting distinct clinical phenotypes and geographic distributions that challenge traditional diagnostic paradigms.

### 3.1 Lipid transfer protein (LTP) syndrome

Lipid transfer proteins are pan‐allergens that cause FIA, particularly in Mediterranean adults and increasingly in pediatric populations globally.^37^, ^101^, ^102^, ^103^ The prototypical allergen Pru p 3 from Rosaceae fruits (peach, apple) exhibit exceptional thermal and proteolytic stability, concentrating in fruit skin.^101^, ^102^ LTP syndrome manifests as polysensitization across phylogenetically unrelated plants, creating broad cross‐reactivity without sensitization hierarchy.^102^ Cross‐sensitization commonly occurs with structurally homologous proteins like Art v 3 (mugwort) and Pla a 3 (plane tree), though these typically remain clinically silent for food reactions while potentially triggering respiratory symptoms.^102^, ^104^, ^105^ Clinical severity demonstrates an inverse relationship with co‐sensitization to profilins or PR‐10 proteins, with Italian cohorts showing 97% nsLTP sensitization rates and significant co‐sensitization patterns (28.4% PR‐10, 5% profilin).^106^

LTP syndrom**e** demonstrates a clear northward expansion across Europe with increasing clinical significance. Mediterranean countries have historically reported high LTP allergy prevalence, but recent surveillance data reveal accelerating clinical allergy and sensitization rates, including pediatric populations. Italian cohort studies document up to 50% increases in LTP sensitization between 2010 and 2020, with walnut (Jug r 3), peanut (Ara h 9), and hazelnut (Cor a 8) LTPs showing the most dramatic rises.^107^ This epidemiological shift extends beyond Southern Europe, with Northern European countries increasingly recognizing LTP allergy as a cause of severe FIA where it was previously rare.^37^, ^100^, ^108^, ^109^, ^110^, ^111^

Tree nuts and peanuts are primary triggers for severe reactions in adults, while tomatoes are notably associated with food‐dependent exercise‐induced anaphylaxis (FDEIA).^105^, ^106^ Pediatric anaphylaxis occurs at a higher rate (46%), often with cofactors like NSAIDs or exercise (27%) and frequent multi‐food reactivity (69%).^100^, ^103^, ^105^, ^112^ Strict food avoidance remains guideline‐endorsed for prevention, though Pru p 3 immunotherapy—limited by adverse effects and insufficient evidence—represents an investigational alternative, with enhanced safety strategies (e.g., allergoids, biologics) under exploration.^113^, ^114^, ^115^

### 3.2 Novel fruits and vegetables allergens

Gibberellin‐regulated protein (GRP) allergies represent a distinct clinical phenotype characterized by high anaphylactic potential and pathognomonic symptoms including facial edema and laryngeal tightness, affecting both children and adults.^99^ GRP sensitization, particularly to Pru p 7 (peach) and Pru m 7 (apricot), demonstrates strong cross‐reactivity within Rosaceae fruits and accounts for severe systemic reactions in 65% of Japanese fruit‐allergic patients who lack concurrent PR‐10 or profilin sensitivities.^116^, ^117^, ^118^ The syndrome exhibits distinctive clinical markers including rapid progression to anaphylaxis, frequent cofactor dependence on NSAIDs or exercise, and characteristic laryngeal symptoms that serve as early predictors of severe reactions, necessitating strict avoidance protocols for cross‐reactive fruits (peach, apricot, cherry, citrus, pomegranate) and emergency preparedness in GRP‐sensitized individuals.^117^, ^119^

Thaumatin‐like protein (TLP) is a class of plant‐derived allergens that constitutes an underrecognized cause of fruit‐induced anaphylaxis, with documented severe systemic reactions to apple (Mal d 2), peach (Pru p 2), banana (Mus a 4), and cherry (Pru av 2) that can progress to life‐threatening anaphylaxis involving respiratory distress, hypotension, and gastrointestinal compromise.^120^, ^121^ Besides fruits, TLPs are also found in wood dust and cereals, potentially triggering respiratory symptoms associated with baker's asthma.^122^ The thermal stability of TLPs enables anaphylactic reactions to processed foods including juices and baked goods, while extensive cross‐reactivity between TLP allergens necessitates broad fruit avoidance in sensitized individuals. Cofactor amplification significantly increases anaphylaxis risk and severity, while diagnostic challenges arise from frequent false‐negative standard allergy tests, requiring component‐resolved diagnostics for accurate identification of TLP sensitization and appropriate adrenaline prescription in this high‐risk population.

## 4 ANAPHYLAXIS WITH UNUSUAL PRESENTATION

### 4.1 Alpha‐Gal Syndrome

Alpha‐gal syndrome (AGS) is a globally significant cause of FIA, triggered by an IgE‐mediated reaction to galactose‐α‐1,3‐galactose (α‐gal), primarily acquired through tick bites.^123^ First linked to cetuximab anaphylaxis and meat allergy in 2007–2009,^124^ AGS uniquely causes delayed allergic reactions (typically 3–6 h) after ingesting mammalian meat or products, ranging from urticaria to anaphylaxis, often with gastrointestinal symptoms.^125^ Co‐factors have also been reported to aggravate the occurrence of anaphylaxis due to AGS.^125^, ^126^, ^127^, ^128^, ^129^, ^130^ Ticks introduce α‐gal from their saliva into humans during bites, inducing IgE production. Subsequent α‐gal ingestion (from meat/products) triggers IgE cross‐linking on mast cells/basophils, causing degranulation. The symptom delay is attributed to the absorption of α‐gal glycolipids via chylomicrons.^131^, ^132^, ^133^

AGS has been reported from every continent except Antarctica.^134^ The US shows a dramatic rise (>6‐fold increase in positive IgE tests from 2011 to 2018; ~96,000 to 450,000 estimated cases since 2010; 20%–31% seroprevalence in the Southeast, linked to *Amblyomma americanum*).^38^, ^135^ Australia reports the highest rate (113/100,000, linked to *Ixodes holocyclus*).^15^ Cases are also documented in Europe, Asia, South America, and Africa.^136^, ^137^, ^138^, ^139^, ^140^ Expanding tick exposure, clinical/diagnostic awareness, and climate change are increasing AGS distribution.^141^

Management of AGS includes strict avoidance of mammalian meat and α‐Gal–containing products, including gelatine, dairy, and certain pharmaceuticals such as vaccines and porcine‐origin heart valves.^142^, ^143^ Tick bite prevention is crucial. While no cure exists, research explores desensitization and anti‐IgE therapies.^144^, ^145^ Increased clinician awareness in endemic areas is vital for timely diagnosis and preventing fatalities.

### 4.2 GOS Anaphylaxis

Galacto‐oligosaccharides (GOS) anaphylaxis represents an unusual presentation of food allergy, characterized by its geographic restriction to Asia, reactions upon first known exposure, and cross‐reactivity with inhalant allergens rather than primary food sensitization. GOS are non‐digestible carbohydrates composed of 2–10 galactose units, with a terminal glucose molecule. GOS are enzymatically synthesized commercially using β‐galactosidase derived from various microorganisms and widely supplemented as prebiotics in dairy products and infant cow's milk formulas.

Although GOS is commonly found in consumer products globally, reports of allergic reactions and anaphylaxis have been confined to Asia–Japan,^146^ Singapore,^35^, ^147^, ^148^ Malaysia, Thailand and the Philippines (unpublished), and Vietnam.^149^ In Singapore, 20 cases of GOS allergy in older children and adults have been documented following the first ingestion of GOS‐containing milk. Clinical manifestations were typical of IgE‐mediated food allergy, with acute symptoms of urticaria, angioedema, rhinorrhea, and wheezing occurring within 30 min in the majority (80%) and not more than 2 h after ingestion. All affected individuals had a positive skin prick test (SPT) and basophil activation test (BAT) to Vivinal GOS (vGOS).

From the screening of a larger atopic cohort, vGOS BAT showed a sensitivity of 83% (95% CI 36%–99%) and specificity of 100% (95% CI 52%–100%) for the diagnosis of GOS allergy^150^ Additionally, subjects with a positive vGOS challenge had larger vGOS SPT wheal sizes (6 mm [3–8 mm], median [range]) compared to those with a negative vGOS challenge (4 mm [0–4 mm]) (*p* < .019). From this cohort, the prevalence of vGOS allergy in the atopic population of Singapore is estimated to be as high as 3.5% (95% CI: 2.2%–5.5%).^150^ The relatively small number of clinical cases observed is likely due to the limited consumption of GOS in Singapore, as the prebiotic supplement is only found in infant cow's milk formula.

Being composed of oligosaccharides, GOS does not induce IgE sensitization. Hence, GOS allergy is likely to arise from cross‐reactive IgE antibodies. The putative primary sensitizers for GOS have been identified as dust mite *Blomia tropicalis* in Singapore and the sea squirt in oyster shuckers in Japan.^35^, ^151^ This phenomenon, characterized by cross‐reactive IgE to GOS with inhalant allergens, explains the symptoms that occur on the very first exposure to GOS, as well as the higher age of presentation (in older children and adults). Over the past few decades, *Blomia tropicalis* allergy has shown a growing prevalence globally, with marked increases reported in South America, as well as East and Southeast Asia.^152^ How this growing prevalence of *Blomia tropicalis* sensitization may influence the occurrence of GOS‐induced anaphylaxis remains to be elucidated. GOS allergy highlights the unique geographical and population differences in food allergy. The development of ‘hypoallergenic’ GOS is an important priority for its safe use in the Asian region.

### 4.3 Co‐Factor Specific Anaphylaxis

#### 4.3.1 Food‐Dependent Exercise‐Induced Anaphylaxis

Food‐dependent exercise‐induced anaphylaxis (FDEIA) is triggered by the combination of physical activity and consuming a particular food; it is not caused by exercise or food alone.^153^ Anaphylaxis triggered by exercise was first described by Maulitz et al.^154^ Kidd et al. reported four such patients and designated the phenomenon of FDEIA.^155^ Recognition of the phenotype spread globally in the 1980s–2000s, with regional patterns emerging.^155^, ^156^ EIA is estimated to represent approximately 2.3%–5% of anaphylactic patients.^157^, ^158^ The prevalence of FDEIA was 0.0046%–0.018% in Japanese children.^156^, ^159^, ^160^

Globally, wheat was the most common causative food, followed by vegetables, seafood, legumes, and fruits.^156^, ^161^, ^162^, ^163^ However, there are regional variations: in Southeast Asia and Latin America, for example, shellfish is the most commonly associated food with FDEIA.^157^ In Japan, shrimp and other crustaceans are the second most common cause of FDEIA after wheat.^156^ Asaumi et al. identified the following as the main causative foods in pediatric cases: wheat alone (25%), a combination of wheat and shrimp (15%), a combination of wheat and apple (10%), and peach alone (10%).^164^

While food antigen‐specific IgE and skin prick tests show insufficient efficiency for FDEIA diagnosis, component‐resolved diagnostics may demonstrate greater utility when available. For example, omega‐5 gliadin‐specific IgE is useful for diagnosing WDEIA in adults.^165^ Food‐exercise provocation tests represent the gold standard for FDEIA diagnosis. The protocol typically involves consuming clinically relevant amounts of suspected food, followed by standardized exercise after 30 min.^84^ If the results are negative, 10 mg/kg aspirin (maximum 500 mg) can be administered 30 min before consuming the food. As demonstrated by Asaumi et al., 20% of positive provocation tests required adrenaline administration, with initial symptoms typically occurring within 30 min of exercise initiation.^164^ Other cofactors related to anaphylaxis are summarized in Table 1.

**TABLE 1:** Anaphylaxis precipitating co‐factors and OIT‐related exercise reactions.

Category	Specific co‐factor	Mechanism/Impact	Key findings/notes	Ref
Food‐dependent Exercise‐induced Anaphylaxis (FDEIA)	Exercise + Specific Food	↓ Gastric acid → intact food proteins ↑ Intestinal permeability → allergen absorption Blood flow redistribution → allergen transport to mast cells Transglutaminase‐enhanced antigen transfer	Triggers: Global: Wheat > vegetables > seafood > legumes > fruit SE Asia/Latin America: Shellfish Japan: Wheat > crustaceans	153, 200, 201
Medications	NSAIDs (incl. Aspirin)	↑ Gastrointestinal permeability → ↑ food allergen absorption	Most clinically significant medication co‐factor	202, 203
	Beta‐blockers	↓ Adrenaline effectiveness → worsens anaphylaxis (blocks adrenergic receptors)	Impairs treatment response during reactions	204, 205
Infections	General infections	Alters immune responses & mucosal barriers (esp. febrile/GI infections)	Triggers reactions to otherwise tolerated foods	^206^
Hormonal factors	Perimenstrual fluctuations	Hormonal changes ↑ anaphylaxis risk	Significant in adolescent girls/women; reactions may occur *only* during specific menstrual phases	^207^
Psychological/Physical	Psychological stress	Neuroendocrine pathways ↓ mast cell stability → ↓ anaphylaxis threshold	Critical in children: academic stress, sleep deprivation, emotional factors ↑ susceptibility	^208^
	Physical fatigue			
Environmental	Heat exposure & high humidity	Acts as adjunct trigger	Particularly relevant in active pediatric populations	^208^
OIT‐related reactions	Wheat OIT	Exercise‐induced allergic reactions on desensitization (EIARDs)	66.7% developed EIARDs *during* OIT; 52.4% maintained risk >5 years post‐OIT	^209^
	Egg OIT		21% developed EIARDs post‐OIT; only 2.1% persisted >5 years	^210^
	Milk OIT			

Abbreviations: EIARDs, exercise‐induced allergic reactions on desensitization; GI, gastrointestinal; NSAIDs, non‐steroidal anti‐inflammatory drugs; OIT, oral immunotherapy.

## 5 MANAGEMENT OF ANAPHYLAXIS

**FIGURE 2:**
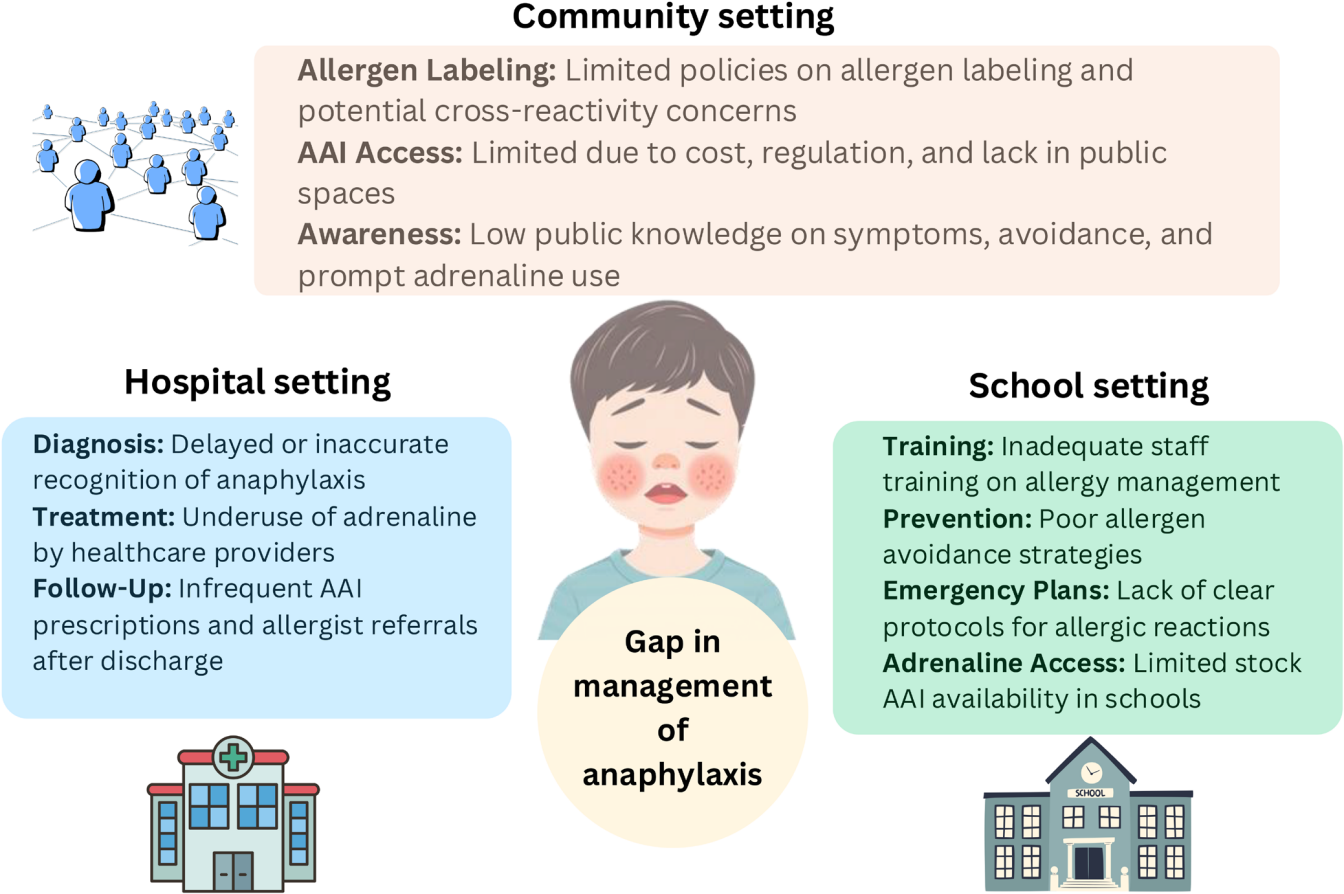
Gaps in the management of anaphylaxis across key settings. This schematic illustrates critical barriers to optimal anaphylaxis care in hospital, school, and community settings. In hospitals, challenges include delayed or missed diagnosis, underuse of intramuscular adrenaline, and poor follow‐up with allergist referrals or AAI (adrenaline auto‐injector) prescriptions. In schools, gaps include inadequate staff training, limited implementation of allergen avoidance strategies, unclear emergency protocols, and restricted access to AAIs. In the community, AAI access remains limited due to cost, regulation, and availability, compounded by low public awareness of anaphylaxis recognition and management. Together, these systemic gaps highlight the need for coordinated, cross‐sector improvements to reduce anaphylaxis‐related morbidity and mortality in children.

Despite advances in immunotherapy and biologic treatments for food allergy, patient education on anaphylaxis action plans and the timely use of adrenaline remains the cornerstone of treatment.^166^, ^167^ This is essential because accidental exposures and adverse reactions—especially during oral immunotherapy—can occur unpredictably and escalate rapidly.^166^, ^168^ In some cases, anaphylaxis may be the first presenting symptom in children with no prior diagnosis of food allergy.^21^, ^169^ Accidental exposures often stem from cross‐contamination during food preparation, mislabeled packaging, undisclosed ingredients, or food sharing among children. While most incidents occur at home, schools, and restaurants are also common settings for allergic reactions.^21^, ^59^, ^170^, ^171^ This section outlines key barriers to effective anaphylaxis management in the community, hospitals, and schools (Figure 2).

### 5.1 Gaps in the Community

Contemporary anaphylaxis management faces critical gaps in addressing emerging and non‐priority allergens that pose significant life‐threatening risks. Non‐priority legumes (lentils, peas, chickpeas) and buckwheat lack mandatory labeling requirements despite documented severe reactions, while sesame labeling remains inconsistent across jurisdictions. Diagnostic challenges arise from complex cross‐reactive syndromes, particularly nsLTP polysensitization and α‐Gal syndrome, which necessitate broad dietary restrictions without curative interventions. Geographic variations in allergen prevalence, including galacto‐oligosaccharides in Asian populations and novel protein sources such as edible insects, create first‐exposure anaphylaxis risks that current prevention models inadequately address. The absence of region‐specific surveillance systems and insufficient community education regarding food‐dependent exercise‐induced anaphylaxis (FDEIA) and tick‐borne α‐Gal syndrome further compound management difficulties. These systematic gaps underscore the urgent need for expanded allergen labeling policies, enhanced cross‐reactivity diagnostic protocols, and targeted regional surveillance to prevent fatal anaphylactic events from increasingly diverse trigger sources.

Adrenaline autoinjectors (AAIs), introduced in the mid‐1990s, are designed to deliver accurate doses of intramuscular adrenaline quickly. However, their availability and accessibility remain uneven globally.^172^, ^173^, ^174^, ^175^ A 2023 survey by the World Allergy Organization found that AAIs were available in only 60% of 43 responding countries—mostly in high‐income regions such as Europe and North America.^176^ This rate was similar to that reported in a 2003–2005 survey of 39 countries, where AAIs were available in 56.4% of the countries surveyed.^172^ Even in countries where AAIs are available, only 16% have national policies ensuring access in public spaces.^176^ In low‐ and middle‐income countries, access is often limited by cost, supply chains, and reliance on imports.^172^, ^174^, ^177^ In the Asia‐Pacific region, AAIs are available in 9 of 13 surveyed countries^178^; however, Bangladesh, Indonesia, Mongolia, and Vietnam lack access. AAI availability in South/Latin America remains critically limited—only 27.8% of South American countries import devices, none manufacture locally, and prohibitive costs (e.g., $180–200 USD per twin‐pack via private import in Brazil) restrict access.^176^, ^179^ Consequently, many nations rely on pre‐filled adrenaline syringes as alternatives.^21^, ^178^

Data from the U.S. national patient registry established by Food Allergy Research and Education (FARE) found that about 50% of individuals with food allergy reported one or more allergic reactions annually. Although most of these reactions were due to unintentional exposure, about 10% resulted from intentional risk‐taking behavior. During such events, 70.2% used antihistamines, 22.8% used AAIs, and 17.6% used corticosteroids.^180^ The APRA registry reported that only 9% of anaphylaxis cases received adrenaline before arriving at a hospital. Even among those who owned AAIs, prehospital use remained low.^21^ Additionally, adrenaline is more often administered in the presence of respiratory symptoms, while non‐respiratory signs (cutaneous or gastrointestinal) are often undertreated.^181^ A meta‐analysis further showed significant underuse of adrenaline in prehospital settings—20.98% in children versus just 7.17% in adults.^182^

Barriers to effective community management include limited access to AAIs due to regulatory and economic constraints, lack of stock AAIs in public areas, and insufficient public education. Increasing awareness of early symptoms, promoting AAI use, and ensuring broader availability are crucial next steps.

### 5.2 Gaps in Hospital Settings

Despite clinical guidelines recommending adrenaline as the first‐line treatment for anaphylaxis, hospital use remains inconsistent. Multiple studies—including APRA data—showed that only 60% of anaphylaxis cases were treated with adrenaline.^21^, ^176^, ^181^, ^183^ Furthermore, many patients are discharged without an AAI prescription or referral to an allergist for follow‐up evaluation.^176^, ^184^ There is also regional and institutional variation in the use of steroids and antihistamines in the acute management of anaphylaxis, with these agents frequently used as adjuncts or, in some settings, as initial therapy before adrenaline, despite consensus that adrenaline is the only first‐line treatment.^185^ Registry data from Canada and Israel^186^, ^187^ show that antihistamines are used prehospital in 44% of cases and corticosteroids in 3%–4%, with similar patterns in emergency departments. Data from APRA also showed that over 60% of anaphylaxis episodes were administered antihistamine and steroids.^21^, ^188^ Fear of administration in children, despite strong safety evidence, may also have played a role in this phenomenon.^189^ This gap overall reflects local practice norms, provider comfort, and patient expectations. A study by Manivannan et al.^190^ demonstrated that implementing an emergency department^191^ order set and making AAIs available in EDs increased adrenaline administration from 33% to 51%. These findings underscore the importance of national clinical guidelines, along with structured implementation in EDs and primary care settings, to ensure timely and accurate diagnosis and treatment.^192^, ^193^

### 5.3 Gaps in Educational Institutions

Schools represent high‐risk environments for allergic reactions—affecting both students with diagnosed allergies and those experiencing their first reactions. Given students' reliance on school staff during emergencies, preparedness is essential.^170^, ^175^, ^194^ The Global Allergy and Asthma European Network (GA^2^LEN) and the European Federation of Allergy and Airways Diseases Patients' Associations (EFA) released a consensus outlining four policy priorities: (1) Training school staff to improve knowledge and confidence; (2) Preventive strategies to reduce accidental allergen exposure; (3) Emergency protocols, including access to adrenaline administration devices; and (4) Promoting inclusivity to reduce isolation and bullying of students with allergies.^170^ Recent 2023 guidelines discourage ineffective school‐wide food bans, instead recommending targeted interventions such as allergen‐restricted zones for vulnerable students and stock AAIs in educational settings.^194^

School and community preparedness for food allergy management varies markedly across global contexts. While national policies for school‐based allergy management are common in Europe and North America, they are currently implemented in only two Asia‐Pacific countries—Australia and Japan.^170^, ^175^ In Western countries, policies are typically supported by robust legislative frameworks such as Sabrina's Law in Canada and Natasha's Law in the United Kingdom, which mandate school‐wide allergy management plans, staff training, emergency action documentation, and transparent allergen labeling for all food services.^170^, ^195^, ^196^ These regulations have been shown to improve consistency with anaphylaxis prevention guidelines and enhance staff readiness to administer adrenaline in emergencies.^170^ By contrast, many Eastern countries lack standardized mandates, and implementation often depends on local advocacy or institutional initiatives.^21^, ^197^ As a consequence, the undertreatment of anaphylaxis with adrenaline observed in previous reports, particularly in children, was partly due to delayed recognition of early symptoms in schools.^21^, ^198^, ^199^ Sociocultural and structural factors—such as lower food allergy awareness, stigma toward medicalized dietary restrictions, and limited access to adrenaline—remain significant barriers to preparedness in several Asian communities. Cultural perceptions that food allergy is exaggerated or uncommon may hinder school engagement and parental disclosure, while language and socioeconomic disparities can further limit educational access and response capacity. Strengthening community‐based education, promoting multilingual training programs, and adapting policy frameworks to local cultural contexts are key steps toward improving equitable preparedness across regions. Bridging this gap will require coordinated efforts involving families, students, school staff, healthcare providers, and policymakers.

## 6 CONCLUSION & FUTURE DIRECTIONS

The evolving anaphylaxis landscape reflects profound shifts in global dietary patterns, environmental exposures, and food production systems that challenge traditional allergen management paradigms. The emergence of non‐priority legumes, edible insects, and region‐specific triggers such as galacto‐oligosaccharides demonstrates that current regulatory frameworks inadequately address novel allergen risks in increasingly interconnected food systems. Complex cross‐reactive syndromes, including LTP polysensitization and tick‐borne α‐Gal syndrome, necessitate sophisticated diagnostic approaches beyond conventional IgE testing to prevent unnecessary dietary restrictions while ensuring patient safety.

Future research priorities must establish comprehensive global surveillance networks modeled on existing registry systems to track emerging allergen patterns across diverse geographic and economic contexts. Development of precision diagnostic tools for atypical presentations, including FDEIA and cofactor‐dependent reactions, represents a critical need for personalized risk stratification. Regulatory harmonization should expand mandatory labeling requirements to encompass novel protein sources and non‐priority allergens while establishing pre‐market allergenicity assessment protocols for emerging foods.

Therapeutic innovation should focus on targeted interventions for complex syndromes, including anti‐IgE therapies for α‐Gal syndrome and desensitization protocols for insect‐derived proteins. Equity‐centered approaches must address disparities in AAI accessibility and implement region‐specific education programs addressing local risk factors such as tick exposure and novel food introductions. This should be complemented by better public education on anaphylaxis reactions and reduced hesitation in pediatric care to achieve the best outcome. Some emerging approaches to improve therapeutic outcomes include digital anaphylaxis action plans, telemedicine for post‐anaphylaxis follow‐up, and simulation‐based training for healthcare providers. Ultimately, effective anaphylaxis management in the 21st century requires collaborative integration of clinical expertise, regulatory oversight, and public health surveillance to anticipate and mitigate risks from our rapidly evolving global food environment.

## AUTHOR CONTRIBUTIONS

**Agnes Sze‐yin Leung:** Conceptualization; writing – original draft; methodology; visualization; writing – review and editing; validation. **Elizabeth Estrada‐Reyes:** Writing – original draft; writing – review and editing. **Kaito Goto:** Writing – original draft; writing – review and editing; validation. **Chiung‐Hui Huang:** Writing – original draft; writing – review and editing; validation. **Janice Min Li:** Writing – original draft; writing – review and editing; validation. **Sowmya Arudi Nagarajan:** Validation; writing – review and editing; writing – original draft. **Thushali Ranasinghe:** Writing – original draft; validation; writing – review and editing. **Sakura Sato:** Validation; writing – review and editing; writing – original draft. **Witchaya Srisuwatchari:** Writing – original draft; validation; writing – review and editing; visualization. **Benjamin Zepeda‐Ortega:** Writing – original draft; validation; writing – review and editing. **Elizabeth Huiwen Tham:** Validation; writing – review and editing; writing – original draft.

## FUNDING INFORMATION

This research received no external funding.

## CONFLICT OF INTEREST STATEMENT

The authors declare no conflicts of interest.

## ETHICS STATEMENT

Not applicable.

## CONSENT STATEMENT

Not applicable.

## Supporting information


**Table S1.** Global variation in age‐related patterns of causative food allergens.

## Data Availability

Not applicable.
