# Supplementary material for: Global trend of food‐induced anaphylaxis: Up to date
Source: Pediatr Allergy Immunol. 2025 Dec 2;36(12):e70246. doi: 10.1111/pai.70246 (PMC12673296; doi:10.1111/pai.70246)
Supplement: Supplementary file 1 — Table S1. Global variation in age‐related patterns of causative food allergens. [file PAI-36-e70246-s001.docx]

Table S1: Global Variation in Age-Related Patterns of Causative Food Allergens

| Region | Registry | Study Period | Study Design | Study Subjects | Age Group | Food Allergens | No. of Patients (% of FIA) | Ref |
| --- | --- | --- | --- | --- | --- | --- | --- | --- |
| Europe, 90 study centers in 10 countries: Germany, France, Switzerland, Ireland, Greece, Austria, Spain, Bulgaria, Italy, and Poland | European anaphylaxis registry (NORA) | July2007- March 2015 | Prospective registry European anaphylaxis registry (Network for Online Registration of Anaphylaxis, NORA) | The study included 1970 children who had experienced anaphylaxis, referred for in-depth diagnosis and counselling.  Severe cases were deliberately oversampled | 0-17 years old | Total  Cashew  Hazelnut  Walnut  Pistachio  Pine nut  Other tree nuts  Celery  Other vegetables  Wheat  Other cereals  Hen’s egg  Cow’s milk  Goat’s milk  Shrimp  Codfish  Other animal products  Peanuts  Soy  Pea  Other legumes  Sesame  Other spices  Fruits | n=1092  n=78 (9)  n=76 (10)  n=42 (7)  n=21 (2)  n=13  n=26 (16)  n=8 (3)  n=11 (3)  n=28 (3)  n=17 (4)  n=120 (14)  n=128 (12)  n=16 (2)  n=18 (7)  n=17 (3)  n=44 (9)  n=297 (28)  n=17 (2)  n=9 (3)  n=35 (2)  n=15 (5)  n=12 (4)  n=36 (5) | ^23^ |
| France | Réseau d’Allergo-Vigilance (RAV) (Allergy-Vigilance network, AVN) | 2002-2020 | Retrospective registry | 25 children with a grade 4 anaphylaxis (Ring and Messmer) out of the 3510 anaphylaxis cases documented in the network | <18 years old | Total  Peanut  Milk  Shrimp  Hazelnut  Wheat  Cashew | n=25  n=10 (40%)  n=7 (28%)  n=1 (4%)  n=1 (4%)  n=0 (0%)  n=1 (4%) | ^26^ |
| Portugal | Registo Portugues de Anafilaxia (Portugues Anaphylaxis Registry) | 2007-2017 | Retrospective registry | 167 patients with anaphylaxis, reported by 58 allergists from all mainland region (Northern, Center, and Southern) and from Azores and Madeira islands, were included in the study | <6 years old | Total  Milk  Tree nuts  Shellfish  Egg  Fresh fruit  Fish  Peanut  Cereals  Seeds  Meats  Others | n=167  n=67 (40%)  n=28 (17%)  n=7 (4%)  n=24 (14%)  n=12 (7%)  n=17 (10%)  n=10 (6%)  n=7 (4%)  n=2 (1%)  n=1 (<1%)  n=2 (1%) | ^27^ |
|  |  |  |  | 138 patients with anaphylaxis, reported by 58 allergists from all mainland regions (Northern, Center, and Southern) and from Azores and Madeira islands, were included in the study | 6-11 years old | Total  Milk  Tree nuts  Shellfish  Egg  Fresh fruit  Fish  Peanut  Cereals  Seeds  Meats  Others | n=138  n=38 (28%)  n=21 (15%)  n=21 (15%)  n=22 (16%)  n=16 (12%)  n=9 (7%)  n=14 (10%)  n=1 (<1%)  n=1 (<1%)  n=0 (0%)  n=4 (3%) | ^27^ |
|  |  |  |  | 106 patients with anaphylaxis, reported by 58 allergists from all mainland region (Northern, Center, and Southern) and from Azores and Madeira islands, were included in the study | 12-17 years old | total  Milk  Tree nuts  Shellfish  Egg  Fresh fruit  Fish  Peanut  Cereals  Seeds  Meats  Others | n=106  n=25 (24%)  n=17 (16%)  n=25 (24%)  n=4 (4%)  n=17 (16%)  n=7 (7%)  n=8 (8%)  n=1 (1%)  n=4 (4%)  n=3 (3%)  n=6 (6%) | ^27^ |
| Finland | Finnish National Anaphylaxis Registry | 2000-2017 | Retrospective registry | 663 children out of 1442 anaphylactic cases based on voluntary reports of systemic allergic reactions filled by hospital personnel and covering the Finnish population of 5.5 million people | <16 years old | Nuts  Milk  Egg  Wheat  Fruits  Cereals  Fish  Seeds  Soya  Crustaceans | n=136 (75.7)  n=114 (17.2)  n=73 (11)  n=72 (10.9)  n=32 (4.8)  n=16 (2.4)  n=13 (2.0)  n=10 (1.5)  n=8 (1.2)  n=3 (0.5) | ^211^ |
| Latin America (67 allergy units involved from 12 Latin-American countries and Spain) | Registro Latino Americano de Anafilaxia (Latin American Anaphylaxis Registry) | July 2018- Dec 2021 | Retrospective registry | 334 children out of a total of 808 patients with anaphylactic reactions | 0-17 years old | Total  Milk  Shellfish  Egg  Tree nuts  Fresh fruits  Fish  Peanut  Mite  Soy  Wheat  Others | n=269 (32.9)  n=104 (38.7)  n=34 (12.6)  n=25 (9.3)  n=23 (8.6)  n=19 (7.1)  n=14 (5.2)  n=11 (4.1)  n=7 (2.6)  n=7 (2.6)  n=5 (1.9)  n=20 (7.4) | ^24^ |
| Brazil | Registro Brasilerio de anafilaxia (Brazilian Anaphylaxis Registry, RBA) | 28^th^June 2021- 15^th^April 2023 | Retrospective registry | 99 children out of 237 Brazilian individuals with a history of anaphylaxis | <18 years old | Total  Cow’s milk  Egg  Peanuts  Wheat  Nuts  Seafood  Fish  kiwi  Others | n=65  n=32 (13.5)  n=13 (5.5)  n=6 (2.5)  n=4 (1.7)  n=2 (0.8)  n=1 (0.4)  n=1 (0.4)  n=0 (0)  n=6 (2.5) | ^31^ |
| Canada | Cross-Canada Anaphylaxis Registry (C-CARE) | April2011- April 2012 | Prospective registry | 168 Children presenting to the Montreal Children’s hospital PED with anaphylaxis were recruited among 81,677 PED visits |  | Total  Peanut  Tree nuts | (87.5)  (29.5)  (15.5) | ^29^ |
|  | Cross-Canada Anaphylaxis Registry (C-CARE) | April2012- April 2013 | Prospective registry | 218 Children presenting to the Montreal Children’s hospital PED with anaphylaxis were recruited among 78,650 PED visits |  | Total  Peanut  Tree nuts | (80.6)  (20.6)  (14.8) |  |
| Canada (British Columbia) | Cross-Canada Anaphylaxis Registry (C-CARE) | April 2011 – May 2020 | Prospective registry | 78 children in British Columbia out of a total of 3096 cases were presented to emergency departments with anaphylaxis | 0-17 years old, median age was 5.2 (IQR, 2.5-9.5) years | Almond  Brazil  Hazel  Cashew  Macadamia  Pecan  Pine  Pistachio  Walnut  Unidentified tree nuts  Multiple with peanut  Multiple without peanut | n=8 (10.3)  n=1 (1.3)  n=13 (16.7)  n=35 (44.9)  n=0 (0)  n=5 (6.4)  n=3 (3.8)  n=3 (3.8)  n=8 (10.3)  n=0 (0)  n=0 (0)  n=2 (2.6) | ^30^ |
| Canada (Ontario and Quebec | Cross-Canada Anaphylaxis Registry (C-CARE) | April 2011 – May 2020 | Prospective registry | 462 children in British Columbia out of a total of 3096 cases were presented to emergency departments with anaphylaxis | 0-17 years old, median age was 5.2 (IQR, 2.5-9.5) years | Almond  Brazil  Hazel  Cashew  Macadamia  Pecan  Pine  Pistachio  Walnut  Unidentified tree nuts  Multiple with peanut  Multiple without peanut | n=31 (6.7)  n=1 (0.2)  n=95 (20.6)  n=142 (30.7)  n=7 (1.5)  n=21 (4.5)  n=19 (4.1)  n=47 (10.2)  n=54 (11.7)  n=17 (3.7)  n=11 (2.4)  n=16 (3.5) | ^30^ |
| England, Scotland, Wales, and Northern Ireland | UK Fatal Anaphylaxis Registry | 1998-2018 | Retrospective | 187 cases of fatal food induced anaphylaxis | 0-15 years old | Unknown  Milk  Peanut  Unidentified nut  Tree nuts  Fish and crustacea  Others | (29)  (26)  (14)  (12)  (9)  (6)  (5) | ^17^ |
| Asia | Asia-Pacific Research Network for Anaphylaxis (APRA) | 2019-2022 | Prospective registry | 16 tertiary centers across Thailand (6), China (8), and Singapore (2) documented 721 anaphylactic episodes in 689 pediatric patients treated or evaluated for anaphylaxis. | 0-3 years old | Total  Eggs  Milk  Peanuts  Tree nuts  Wheat  Others  Fish  Shellfish  Unknown  Soy | n=260  n=82 (31.54)  n=58 (22.31)  n=32 (12.31)  n=33 (12.69)  n=20 (7.69)  n=18 (6.92)  n=6 (2.31)  n=6 (2.31)  n=4 (1.54)  n=1 (0.38) | ^21^ |
|  |  |  |  |  | 4-6 years old | Total  Tree nuts  Shellfish  Others  Milk  Peanut  Egg  Wheat  Unknown  Fish  Soy | n=66  n=18 (27.27)  n=12 (18.18)  n=11 (16.67)  n=8 (12.12)  n=8 (12.12)  n=4 (6.06)  n=3 (4.55)  n=2 (3.03)  n=0 (0)  n=0 (0) | ^21^ |
|  |  |  |  |  | 7-12 years old | Total  Shellfish  Peanut  Tree nut  Others  Milk  Wheat  Fish  Egg  Soy  Unknown | n=62  n=23 (37.10)  n=10 (16.13)  n=9 (14.52)  n=9 (14.52)  n=5 (8.06)  n=3 (4.84)  n=2 (3.23)  n=1 (1.61)  n=0 (0)  n=0 (0) | ^21^ |
|  |  |  |  |  |  | Total  Shellfish  Peanut  Others  Milk  Tree nuts  Wheat  Fish  Soy  Unknown  Egg | n=118  n=48 (40.68)  n=22 (18.64)  n=22 (18.64)  n=6 (5.08)  n=6 (5.08)  n=6 (5.08)  n=4 (3.39)  n=2 (1.69)  n=2 (1.69)  n=0 (0) | ^21^ |
| Singapore | Multicentre, observational study | 2015 - 2022 | Retrospective | 1188 cases of childhood anaphylaxis visited the emergency departments | 0-19 years old | Total  Peanut  Tree nuts  Cashew  Walnut  Egg  Milk  Shellfish  Wheat  Fish  Sesame  Soy  Kiwi  Coconut  Bird nest  Galacto-oligosaccaride | n=1188  n=110 (9.3)  n=105 (8.8)  n=54 (4.5)  n=16 (1.6)  n=123 (10.4)  n=95 (8.0)  n=93 (7.8)  n=20 (1.7)  n=13 (1.1)  n=4 (0.3)  n=3 (0.3)  n=5 (0.4)  n=4 (0.3)  n=2 (0.2)  n=1 (0.1) | ^34^ |
| China, Wuhan | Tongji Hospital's Data Platform Application Portal (DPAP) across its three Wuhan campuses: Sino-French New City (Caidian), Qiaokou (Hankou), and Optics Valley (East Lake High-Tech Zone) | Jan 1 2003 – Dec 31 2023 | Prospective registry | 362 anaphylaxis patients out of 2,139,272 inpatients, with 362 recorded cases in the data base | 0-3 years old | Total  Unspecified  Seafood  Grain  Dairy products  Eggs  Fruits  Vegetables  Meat  Soy products  Health supplements | (28.6)  (7.1)  (14.3)  (7.1)  (35.7)  (0.0)  (0.0)  (7.1)  (7.1)  (14.3)  (7.1) | ^25^ |
|  |  |  |  |  | 4-6 years old | Total  Unspecified  Seafood  Grain  Dairy products  Eggs  Fruits  Vegetables  Meat  Soy products  Health supplements | (21.1)  (25.0)  (50.0)  (0.0)  (0.0)  (25.0)  (0.0)  (0.0)  (0.0)  (0.0)  (0.0) | ^25^ |
|  |  |  |  |  | 7-12 years old | Total  Unspecified  Seafood  Grain  Dairy products  Eggs  Fruits  Vegetables  Meat  Soy products  Health supplements | (6.5)  (100.0)  (0.0)  (0.0)  (0.0)  (0.0)  (0.0)  (0.0)  (0.0)  (0.0)  (0.0) | ^25^ |
|  |  |  |  |  | 13-18 years old | Total  Unspecified  Seafood  Grain  Dairy products  Eggs  Fruits  Vegetables  Meat  Soy products  Health supplements | (0.0)  (0.0)  (0.0)  (0.0)  (0.0)  (0.0)  (0.0)  (0.0)  (0.0)  (0.0)  (0.0) | ^25^ |
| Korea | Multicenter anaphylaxis registry in Korea | Nov 2016 – Dec 2018 | Retrospective registry | 335 children out of a total of 558 participants from 16 centers in Korea were registered | <18 years old | Total  Hen’s egg  Cow’s milk  Walnut  wheat  Peanut  Kiwi  Pine nut  Buckwheat  Soybean  Others | 284  n=72 (25.4)  n=51 (18.0)  n=27 (9.5)  n=23 (8.1)  n=14 (4.9)  n=12 (4.2)  n=11 (3.9)  n=9 (3.2)  n=5 (1.8)  n=60 (21.1) | ^32^ |
| Japan | Multicenter Japanese Anaphylaxis Registry (Japanese Society of Allergology) | Feb 2015 - Oct 2017 | Prospective registry | 767 anaphylaxis emergency presentation at training and teaching facilities of the Japanese Society of Allergology. | Median age: 6, interquartile range: 3–21 years, range 0-100 years | Total  Hen’s egg  Wheat  Peanut  Walnut  Fish  Fish egg  Fruits  Buckwheat  Soy  Crustaceans  Mollusca  Macadamia nut  Almond  Barley  Butterbur scape  Others  Unknown | n= 522  n= 112 (22)  n=103 (20)  n=65 (12)  n=21 (4)  n=18 (3)  n=17 (3)  n=16 (3)  n=11 (2)  n=11 (2)  n=11 (2)  n=6 (1)  n=5 (1)  n=2 (0.4)  n=2 (0.4)  n=2 (0.4)  n=5 (1)  n=65 (13) | ^33^ |
| Japan | Comprehensive survey of pediatric anaphylaxis done by Aichi Medical Association | Apr 2016 – Mar 2020 | Retrospective | 3424 anaphylaxis cases from 87 secondary and 25 tertiary emergency care hospitals in Aichi Prefecture are identified | <15 years old | Total  Egg  Milk  Wheat  Tree nuts  Peanuts  Fish roe  Fruits  Crustacean  Buckwheat  Fish  Soybean  Others  Multiple  Unspecified | n=1948  n=378 (19.4)  n=339 (17.4)  n=171 (8.8)  n=208 (10.7)  n=109 (5.6)  n=69 (3.5)  n=48 (2.5)  n=32 (1.6)  n=29 (1.5)  n=21 (1.1)  n=17 (0.9)  n=55 (2.8)  n=150 (7.7)  n=322 (16.5) | ^85^ |
| USA | Observational study using a national administrative claims database | 2005-2014 | Retrospective | This study analyzed time trends in FIA among patients <18 years requiring emergency care or hospitalization | 0-2 years | Peanut Tree nuts/Seeds  Fruit/Vegetables Unspecified (No. of FIA ED visits per 100,000 enrollees) | 3.5 - 8.4 2.7 - 4.2 1.5 - 4.5 3.2 - 6.1 | ^28^ |
|  |  |  |  |  | 3-6 years | Peanut Tree nuts/Seeds  Fruit/Vegetables Unspecified | 3.3 - 6.5 2.0 - 6.4 1.7 - 3.5 2.2 - 6.4 |  |
|  |  |  |  |  | 7-12 years | Peanut Tree nuts/Seeds  Fruit/Vegetables Unspecified | 1.4 - 4.3 1.0 - 3.8 0.7 - 3.6 1.6 - 4.7 |  |
|  |  |  |  |  | 13-17 years | Peanut Tree nuts/Seeds  Fruit/Vegetables Unspecified | 0.9 - 6.0 0.6 - 3.5 0.8 - 3.8 0.9 - 5.6 |  |

Reference:

211. Edelman SM, Kukkonen AK, Mäkelä MJ. Eliciting allergens and treatment of anaphylaxis: Report of the finnish national anaphylaxis registry. Allergy. 2019;74(10):2010-3. doi:10.1111/all.13858.
